# Retroauricular squamous cell carcinoma developing on a burn scar: Marjolin's ulcer a case report and review of the literature

**DOI:** 10.1016/j.ijscr.2025.111183

**Published:** 2025-03-19

**Authors:** Alia Methneni, Chaima Ben Ammar, Sawssen Dhambri, Skander Kedous

**Affiliations:** Head and Neck Surgery Department, Salah Azaez Oncology Institute, Tunis, Tunisia

**Keywords:** Marjolin's ulcer, Clinical features, Outcome, Burn scar, Case report

## Abstract

**Introduction and importance:**

Marjolin's ulcer is a cutaneous malignancy that arises in the setting of previously injured skin. The predominant histological type remains squamous cell carcinoma. The purpose of this article was to discuss, through our case, clinical and anatomopathological features of Marjolin's ulcer as well as its treatment options and prognosis. Our case is accompanied by a comprehensive review of the literature.

**Case presentation:**

We report a case of cutaneous squamous cell carcinoma that developed after fourty years of a burn. A histopathological examination of the ulcer concluded that it is a well-differentiated squamous cell carcinoma. The patient underwent surgical excision of the tumor with latissimus dorsi flap reconstruction and lymph node dissection, followed by adjuvant radiotherapy. After six months, a flap weaning was carried out with good functional progress. Our work has been reported in line with the SCARE criteria.

**Clinical discussion:**

Marjolin's ulcer has a relatively low incidence and often occurs as a rare complication of chronic wounds, burn scars, or other types of tissue damage. It should be suspected in the presence of delayed healing of a wound that becomes exuding and malodorous. To date there is no consensus on the therapeutic management of Marjolin's ulcer. According to data literature, surgical intervention stands as the primary therapeutic modality.

**Conclusion:**

Marjolin's ulcer is a rare but aggressive malignancy that can arise decades after skin injury, as illustrated by our case. The delayed onset and potential for late diagnosis emphasize the need for vigilance in chronic wound follow-up.

## Highlights

• Marjolin's ulcer (MU) is a cutaneous malignancy that arises in the setting of previously injured skin
• Marjolin's ulcer should be suspected in the presence of delayed healing of a wound that becomes exuding and malodorous
• the diagnosis remains histological
• management is not consensual : surgery, radiotherapy, Chemotherapy
• The prognosis is poor with high risk of recurrences.

## 1 Introduction

Marjolin's ulcer manifests as a cutaneous malignancy arising in areas of previously compromised skin, including chronic wounds, venous stasis ulcers, lupus vulgaris, pressure sores, and exposure to radiotherapy [1]. It has a ubiquitous distribution across the cutaneous surface. This condition, considered rare, exhibits an estimated incidence ranging between 1 % and 2 % among individuals with burn scars [2].

Originating in the 19th century, Jean-Nicholas Marjolin documented the inaugural case of Marjolin's ulcer [1]. Currently, a comprehensive understanding of its pathophysiology remains partially elusive. Histologically, the prevailing subtype is squamous cell carcinoma (SCC) [2]. The imperative for timely diagnosis and expeditious surgical intervention, coupled with adjunctive therapy, is underscored by the heightened risk of metastases and potential compromise to vital organ structures.

Our aim is to discuss, through our case, clinical and anatomopathological features of MU as well as its treatment options and prognosis. This work has been reported in line with the SCARE criteria.

## 2 Case report

**Fig. 1:**
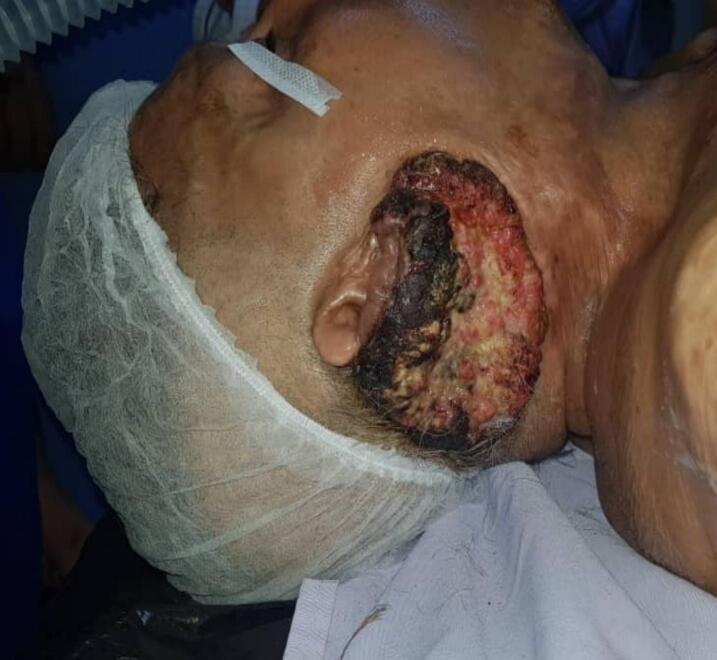
Ulcerating and granulating retroauricular lesion.

This is a 64-year-old female patient with a history of third-degree burns in 1981 involving the neck, chest, and ear region, which required a skin graft taken from the right thigh and has shown good progress since then. She was referred to the ENT, Head, and Neck Surgery Department at the Salah Azeize Institute in January 2018. She recently presented with an ulcerating and granulating lesion progressing for two years and measuring ten centimeters in its largest dimension, located in the right retroauricular area. A large amount of exudate was observed on the wound, and a foul odor was present (Fig. 1). The patient was followed up in a primary care center and despite prolonged antibiotic therapy and regular dressing changes, the ulcer failed to heal.

A biopsy was performed, revealing a well-differentiated, keratinizing squamous cell carcinoma infiltrating the dermis. A computed tomography (CT) scan (cervico-thoraco-abdomino-pelvic) was requisitioned, revealing a tumor infiltrating the skin, parotid gland, sternocleidomastoid muscle, and the wall of the external auditory canal. The CT scan indicated dedifferentiated infracentimetric intraparotid and right level II lymph nodes.

**Fig. 2:**
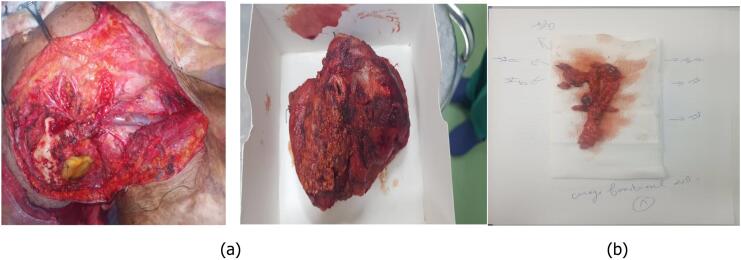
a: Excision of the tumor + a subtotal parotidectomy + exclusion of the external auditory canal. b: Right cervical lymph node dissection.

**Fig. 3:**
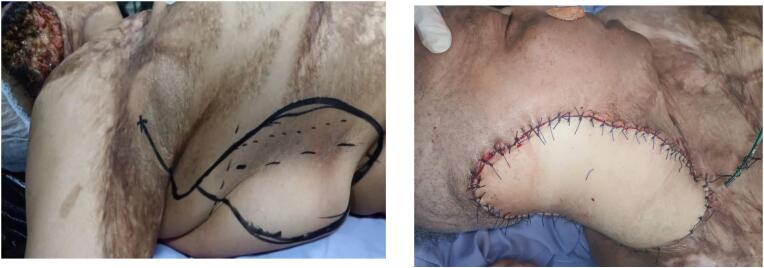
Reconstruction of the tissue loss using a musculocutaneous flap based on the right latissimus dorsi.

The case was discussed in a multidisciplinary committee, and it was decided to proceed with tumor excision surgery and lymph node dissection, followed by adjuvant radiotherapy. The tumor did not extend to the bone, so neither a mastoidectomy nor a bone resection was necessary. The invasion was limited to the soft tissues, allowing for complete excision without the need for osseous intervention. Additionally, the middle ear mucosa and adjacent structures were not involved, and therefore, no specific surgical management was required for these areas. Our patient underwent surgical excision of the tumor involving the right pinna, a subtotal right parotidectomy with surgical obliteration of the external auditory canal (Fig. 2a), and homolateral right modified radical neck dissection type III (levels I-V) (Fig. 2b). The reconstruction of the tissue loss was performed using a musculocutaneous flap based on the right latissimus dorsi muscle, which is pedicled to the thoracodorsal artery (Fig. 3).

The definitive pathology report concluded that it was a well-differentiated, keratinizing squamous cell carcinoma infiltrating the dermis with intact margins of the external auditory canal, uninvaded excision limits, and no vascular emboli or perineural invasion. The cervicoparotid lymph node dissection revealed metastasis in four out of eight lymph nodes (4 N+/8 N), with no capsular rupture, thus, only radiotherapy has been indicated without concurrent chemotherapy.

After six months, a flap weaning was carried out with good functional progress. The patient's last consultation was in November 2023, with no tumor recurrence and a favorable local condition. A canaloplasty and autopoiesis were proposed, but the patient was lost to follow up.

## 3 Discussion

### 3.1 Strengths and limitations in our approach to this case

#### 3.1.1 Strengths

Our case was discussed in a multidisciplinary committee, ensuring a comprehensive evaluation and treatment plan. The patient underwent extensive surgical management, including the excision of the tumor involving the right pinna, subtotal right parotidectomy, and homolateral right cervical lymph node dissection, which addressed the full extent of the disease. To manage the tissue loss, a reconstruction was performed using a musculocutaneous flap based on the right latissimus dorsi muscle, showcasing our team's expertise in advanced reconstructive techniques.

#### 3.1.2 Limitations

The patient presented with an ulcerating lesion that had been progressing for 2 years, indicating a delayed presentation and potential delays in diagnosis and treatment initiation. Although there was an initial favorable response to treatment, the patient was lost to follow-up after undergoing canaloplasty and autopoiesis. This limited follow-up may impact long-term monitoring and management, increasing the risk of undetected recurrences or complications.

### 3.2 Etiology and characteristics of Marjolin's ulcer

Marjolin's ulcer has a relatively low incidence and often occurs as a rare complication of chronic wounds, burn scars, or other types of tissue damage. The exact incidence may vary depending on the population studied and the underlying medical conditions that predispose individuals to developing Marjolin's ulcer [1,2]. Since 1865, when Jean Nicolas reported the first case of Marjolin's ulcer, 574 cases have been recorded to date [3]. In 2019, Fei Xiang et al. reported the highest number of cases, documenting 140 patients with Marjolin's ulcer [4]. The cervicofacial region represents the second most common site, accounting for 34.5 % of cases [5].

Originally, the term “Marjolin's ulcer” specifically referred to squamous cell carcinoma resulting from the malignant transformation of post-burn ulcers. However, the contemporary understanding of Marjolin's ulcer includes all malignant tumors that develop within ulcers [3,5]. The predominant histological type observed in Marjolin's ulcers is squamous cell carcinoma (on the lower limbs and the head and neck), basal cell carcinoma and melanoma being the subsequent most frequently identified types [5].

Carcinogenesis in the context of chronic wounds is driven by a complex interplay of persistent inflammation, progressive genetic alterations, and a tissue microenvironment conducive to malignant transformation. Chronic inflammation resulting from a non-healing wound or unstable scar tissue generates prolonged oxidative stress and continuous release of pro-inflammatory cytokines such as TNF-α, IL-6, and IL-1β, which promote uncontrolled cell proliferation and mutagenesis. This persistent inflammatory microenvironment can lead to genomic instability and epigenetic modifications, such as hypermethylation of tumor suppressor genes or aberrant activation of oncogenic pathways, particularly those involving p53 and EGFR. In our case, the 40-year latency period suggests a slow but progressive accumulation of these molecular alterations, possibly modulated by individual factors such as immune system efficacy, the degree of vascularization within the scar tissue, and potential environmental exposures (e.g., smoking, chronic infections, ultraviolet radiation). One possible hypothesis is that the initial burn scar maintained relative tumor quiescence for decades before a triggering event—such as trauma or age-related decline in immune surveillance—facilitated progression to an invasive malignancy.

Based on existing literature, the pathogenesis of a Marjolin ulcer remains ambiguous, with several theories proposed. Presently, the prevailing theories include the inflammatory irritation hypothesis and foreign body reaction triggered by the implantation of traumatic epithelial elements. Indeed, chronic tissue irritation causes toxins release because of poor vascularization and autolysis of scar tissue [6]. It has also been theorized that chronic ulcers provide an environment conducive to cancer growth because of reduced blood flow, compromised immune response, and the progressively poorer quality of regenerated epithelial tissue [7].

However, recent research suggests that Marjolin's ulcers are primarily due to epigenetic factors. In their research, Harland et al. detected a homozygous deletion of the p53 gene in burn-related carcinoma [6], while Lee et al. identified Fas gene mutations in regions essential for apoptosis in certain burn scar-related squamous cell carcinomas [7].

### 3.3 Clinical features of Marjolin's ulcer

According to the literature, Marjolin's ulcer should be suspected in the presence of delayed healing of a wound that becomes exuding and malodorous. Its age of onset varies between fifteen and eighty-five years, with a gender ratio of 3:1 [6,7]. To rule out a primary cancer as the cause of an ulcer, there is typically a minimum required duration of the lesion, set at 11.3 years (range, five months to fourty-eight years) and nineteen years (range, eleven to thirty-eight years). In the two case series of thirty-nine and fifty-six patients, respectively [8]. In accordance with the literature, our patient had been experiencing the ulcer for a duration of twenty years. The diagnosis is histological, resting on multiple deep biopsies taken from the center and peripheral margins of the lesion.

The main imaging features of Marjolin's ulcer are bone destruction, soft tissue mass, periosteal reaction, and irregularity of the boundaries of the lesion and adjacent soft tissues. All of which were confirmed on the patient's CT scan. MRI is requested as part of the staging evaluation, and it is particularly useful for delineating perineural extension [7,8]; however, the diagnostic gold standard remains the PET scan. In our case, the MRI and the PET scan were not requested.

Distinguishing between bone destruction and periosteal reaction observed on imaging as a result of inflammatory infection versus tumor cell invasion can be challenging. Consequently, suspected bone involvement may warrant excision to prevent recurrence attributed to residual tumors. In his study, xiang et Al identified among the 46 patients with bone invasion, twenty-five had invasion of the skull bones, highlighting the skull's susceptibility to Marjolin's ulcer invasion [6]. Scalp ulcers from Marjolin's ulcer are difficult to excise completely and often exhibit a high recurrence rate. Additionally, given the heightened surgical risk associated with skull tissue invasion, a larger percentage of patients with skull invasion may opt against surgery [4]. In our case, we did not observe any invasion of the scalp in the imaging data.

Marjolin's ulcer is highly lymphophilic, with lymph nodes being the primary site of metastasis; Sentinel lymph node biopsy offers a minimally invasive approach to assess lymph node metastasis in patients with squamous cell carcinoma-type Marjolin's ulcers [8].Therefore, PET-CT findings alone may not suffice for definitive diagnosis of lymph node metastasis and should be complemented by ultrasound-guided biopsy results [3]. For our patient, an ultrasound was not requested; instead, a CT scan was requested as the first-line imaging modality. The PET scan was not requested due to the patient's financial constraints and its unavailability at the hospital.

### 3.4 Treatment and prognosis

To date there is no consensus on the therapeutic management of Marjolin's ulcer. According to data literature, surgical intervention stands as the primary therapeutic modality [3].

All authors have concurred on the necessity of an extensive resection of the tumor. Regarding the safety margins during excision, they should exceed two centimeters (ranging from 2.5 to 5 cm in accordance with the size of the lesion and its location) [9]. The resection depth is dictated by the extent of tumor cells invasion, which can reach the deep tissues or even the bones, complicating the achievement of radical excision of the lesion [4].

Based on literature data, in the presence of nodal involvement, lymph node dissection is systematically performed. In the absence of clinically or radiographically identified lymphadenopathy, the consideration of preventive dissection is subject to discussion [9]. Indeed, certain authors favor systematic prophylactic lymph node dissection, due to the notable incidence of identified nodal metastases [6,7]. Furthermore, other recent studies have concluded a low rate of nodal metastases, not justifying systematic prophylactic dissection [8,9]. Thus, it would be reasonable that the decision for lymph node dissection should be personalized and take into account the tumor's location, degree of differentiation, size, depth of invasion, and the presence of clinically or radiologically identifiable lymph nodes. In this case, the patient's history of burns necessitated a more extensive surgical approach, including wider excision margins to ensure complete removal of the tumor. Additionally, given MU's higher propensity for lymphatic spread, a lower threshold for lymph node dissection was considered. Finally, the need for closer and prolonged follow-up was emphasized, as MU carries a higher risk of local recurrence compared to standard SCC.

After the excision of Marjolin's ulcer, wound repair and functional reconstruction are key to improving life quality. It is generally considered that skin graft repair should be used as much as possible. If bones and tendons are exposed, skin flap repair should be performed. Local skin flap repair should be used if possible, while island skin flap graft repair is the second most desirable alternative. Free skin flap repair should not be the common method of choice [10].

The prognosis depends on several factors, including the presence or absence of metastases at the discovery of the tumor, size, and degree of differentiation. In general, the prognosis is poor with high risk of recurrences. Several studies have indicated that Marjolin's ulcer tends to recur relatively quickly [[8], [9], [10]]. Among recurrent cases, the majority (68.1 %) experience a relapse within 1 year following surgery, indicating that the first year post-surgery is the critical period for recurrence [11]. This necessitates, in all cases, long-term periodic follow-up due to the risk of recurrence and distant metastases (cerebral, hepatic, pulmonary, and renal) [6,8,10]. In our case, there has been no recurrence or distant metastasis revealed up for five years.

Due to the high recurrence rate and potential for metastasis, adjuvant treatments are often considered to improve outcomes. Chemotherapy has not been established as an effective adjuvant treatment for Marjolin's ulcer in the literature. Furthermore, the efficiency of intra-arterial methotrexate infusions for SCC remains uncertain and requires further investigations [10].

However, radiotherapy holds a significant place in the management of Marjolin's ulcers. According to the majority of series, it stands as adjuvant treatment in cases where the malignancy presents as poorly differentiated, high-grade lesions (grade III), and lesions larger than 10 cm in diameter. Additionally, when the malignancy has infiltrated the bone or when there are distant metastases and regional lymph node metastases. Radiotherapy is the primary treatment for Marjolin's ulcers in cases deemed inoperable. It can help to reduce the size of the tumor, manage symptoms, and improve local control of the disease, which is crucial given the aggressive nature of Marjolin's ulcers and their propensity for recurrence [7]. Nevertheless, some authors raise concerns about the efficacy of radiotherapy, attributing their doubts to the reduced vascularization surrounding the ulcerated tissue, which may impede the treatment's effectiveness [10]. In the Chinese series by Xiang et al., involving one hundred and fourty patients with Marjolin's ulcer, all post-operative patients with fully healed wounds were advised to undergo local radiotherapy. However, only a small proportion of these patients opted for the treatment. Additionally, patients who did not undergo surgery were not recommended for radiotherapy due to the presence of ongoing wound healing [8]. In our case, the cervicoparotid lymph node dissection revealed metastasis in four out of eight lymph nodes (4 N+/8 N), with no capsular rupture, thus, only radiotherapy has been indicated without concurrent chemotherapy.

In the literature, the reported five-years survival rate ranges from 40 to 69 % [10], thus the preventive component stands as a cornerstone in the management of Marjolin's ulcer, playing a pivotal role in preventing the onset of the tumor. Thus, raising awareness among primary care physicians regarding recurrent or chronic ulcers that warrant excision even in the absence of malignancy, as well as emphasizing the importance of appropriate wound management.

## 4 Conclusion

In conclusion, Marjolin's ulcer lesions are aggressive and carry a poor prognosis with a high rate of recurrence Therefore; any suspicious non-healing or ulcerative lesion that arises in a chronic scar should undergo biopsy to aid in the early identification and appropriate disease management. This case allows a remind for healthcare professionals of the importance of proper scar management and give information that may prop in the establishment of clinical practice guidelines for the opinion and treatment of Marjolin's ulcer.

## Author contribution

• The following authors were directly involved in the patient's care: Alia.Methneni, Chaima.Ben Ammar
• The following authors were not directly involved in the patient's care, they contributed to the manuscript by: Sawssen.Dhambri, Skander.Kedous

## Consent

Written informed consent was obtained from the patient for the publication of their clinical details and images.

## Ethical approval

The local ethical committee of our institute approved the publication of this study (Decision number: 20/2019/LIRB/ [acceptance of local institutional board]).

The research was conducted ethically, with all study procedures being performed in accordance with the requirements of the World Medical Association's Declaration of Helsinki.

## Guarantor

Chaima Ben Ammar.

## Funding

The authors declared that no grants were involved in supporting this work.

## Conflict of interest statement

All other authors declare no conflict of interest.

## Data Availability

Data used during this study are available from the corresponding author on reasonable request.
